# The allocation and fairness of health human resources in Chinese maternal and child health care institutions: a nationwide longitudinal study

**DOI:** 10.1186/s12913-023-09076-5

**Published:** 2023-02-13

**Authors:** Yuan Ma, Pei Xiao, Li Yu, Henfan Ni, Shiyao Huang, Meixian Wang, Yuxiang Huang, Li Li, Lian Yang, Chunjian Tan, Zhigang Zhong

**Affiliations:** 1 Department of Medical Record Management, West China Second University Hospital, Chengdu, Sichuan, China; 2 Key Laboratory of Birth Defects and Related Diseases of Women and Children (Sichuan University), Ministry of Education, Chengdu, Sichuan China; 3 Medical Insurance Office, West China Fourth Hospital, Sichuan University, Chengdu, Sichuan China; 4 Department of Pharmacy, West China Hospital, Sichuan University, Chengdu, Sichuan China; 5 Chinese Evidence-based Medicine Center, West China Hospital, Sichuan University, Chengdu, Sichuan China; 6 NMPA Key Laboratory for Real World Data Research and Evaluation in Hainan, Chengdu, Sichuan China; 7 National Center for Birth Defects Monitoring, West China Second University Hospital, Sichuan University, Chengdu, Sichuan China; 8 School of Public Health, Chengdu University of Traditional Chinese Medicine, Chengdu, Sichuan China; 9 Department of Quality Control and Evaluation, Chengdu Third People’s Hospital, Chengdu, Sichuan China; 10 Department of Prevention, Office of Cancer Prevention and Treatment, Sichuan Cancer Hospital & Institute, Sichuan Cancer Center, Cancer Hospital Affiliate to University of Electronic Science and Technology of China, 610041, Chengdu, Sichuan, China

**Keywords:** Maternal and child health care institutions, Health human resources, Allocation status, Fairness assessment (FA), China

## Abstract

**Background:**

In response to an aging population, the Chinese government implemented the three-child policy in 2021 based on the comprehensive two-child policy. With the implementation of the new birth policy, people’s maternal and child health (MCH) needs will also increase. The allocation and fairness of MCH human resources directly affect people’s access to MCH services. The purpose of this study is to analyze the allocation of health human resources in Chinese maternal and child health care institutions, evaluate the fairness of the allocation, to provide a reference for the rational allocation of MCH human resources.

**Methods:**

The data of health technicians, licensed (assistant) physicians, and registered nurses in maternal and child health care institutions nationwide from 2016 to 2020 were included. The health resource density index (HRDI) is used to evaluate the allocation level of MCH human resources. The Gini coefficient (G) and Theil index (T) are used to evaluate the fairness of the allocation of MCH human resources from the perspectives of population and geographic area.

**Results:**

From 2016 to 2020, the average annual growth rate of the number of health technicians, licensed (assistant) physicians, and registered nurses in Chinese maternal and child health care institutions was 7.53, 6.88, and 9.12%, respectively. The Gini coefficient (G) of the three types of MCH human resources allocated by population were all below 0.23, and the Gini coefficient (G) allocated by geographical area were all above 0.65. The Theil index (T) of the three types of MCH human resources allocated by population was all lower than 0.06, and the Theil index (T) allocated by geographical area was all higher than 0.53. In addition, the three types of MCH human resources allocated by population and geographic area contributed more than 84% of the Theil index within the group (T_*intra*_) to the Theil index (T).

**Conclusions:**

China’s MCH human resources were fair in terms of population allocation, but unfair in terms of geographical area allocation. In the future, more attention should be paid to the geographical accessibility of MCH human resources, and the allocation of resources should comprehensively consider the two factors of serving the population and geographical area.

## Background

The World Health Organization (WHO) has always regarded maternal and child health (MCH) as a priority area of healthcare security [1]. As the world’s most populous country, China also has a large population of women and children. Actively and effectively doing a good job in MCH is an important task of the Chinese government, which has important strategic significance for improving the health level of the whole people and promoting the construction of a healthy China [2].

The Chinese government has always attached great importance to the health of women and children. It has successively promulgated the Law on MCH, the Law on the Protection of Women’s Rights and Interests, the Law on the Protection of Minors, the Outline for the Development of Chinese Women, and the Outline for the Development of Chinese Children, etc., and has continuously improved the legal system and policy system for MCH [3–5]. On the other hand, the implementation of the national basic public health service project, the sinking of MCH high-quality resources, the construction of MCH service networks, the increasing investment in maternal and child health institutions, and the continued establishment of the MCH service system, are all aimed at improving the fairness and accessibility of MCH services [4, 6]. Based on these series of measures, China’s MCH care has made remarkable achievements. The maternal mortality rate has dropped from 1500/100,000 before 1949 to 16.9/100,000 in 2020, and the infant mortality rate has dropped from 200‰ before 1949 to 5.4‰ in 2020 [7, 8]. All in all, the Chinese government has made great efforts in the field of MCH, and the rights to survival and health of Chinese women and children have been fully guaranteed.

At this stage, the population of all countries in the world has an aging development trend, which is also China’s basic national condition. In the 1970s, to control population growth and improve the quality of the population, the Chinese government launched a nationwide family planning program [9]. This plan has achieved the expected goals well, but on the other hand, it has also led to an aging structure. According to China’s seventh census bulletin, the country’s total population was 1.412 billion, of which 191 million were aged 65 and above, accounting for 13.50% [10]. China’s population structure is rapidly advancing in the direction of aging and is about to enter a deeply aging society. To actively cope with this situation, the Chinese government fully implemented the two-child policy in 2015, that is, a couple can have two children [11]. On this basis, the Chinese government promulgated the three-child policy in 2021, that is, a couple can have three children [12]. From a strategic perspective, the new birth policy is conducive to improving the country’s population structure, expanding the supply of labor, maintaining the advantages of human resource endowments, and thus promoting stable economic and social development [13]. It is worth mentioning that after the adjustment of the birth policy, people’s demand for MCH services will increase to a certain extent. However, health resources are scarce. So in the new period, it is particularly important to allocate the limited MCH resources fairly and reasonably to ensure that people’s needs for MCH services are met.

Existing studies on equity have mostly focused on other types of health resources, such as primary health care resources [14], traditional Chinese medicine health resources [15], public health facilities [16, 17], and emergency medical services [18], etc. Several of the studies on health human resources were focused on regional overall health human resources [19–22], and few studies have been conducted on women and children’s institutions. In addition, most of the studies preferred to divide China into three regions according to geographic affiliation to discuss the issue of fairness [23, 24]. However, this method of division could not clarify the impact of regional economic differences on the fairness of resource allocation. Therefore, based on the data on health human resources in China’s maternal and child health care institutions during the 13th Five-Year Plan period (2016-2020), this study divides different regions according to the level of regional per capita Gross Domestic Product (GDP) and explores the allocation and fairness of MCH human resources. The research aims to provide decision-making reference for the Chinese government to optimize the allocation of MCH human resources during the 14th Five-Year Plan period, to achieve fair and accessible MCH services as a whole, and to contribute to the construction of a healthy China.

## Methods

### Data source

The data on health human resources (health technicians, licensed (assistant) physicians, and registered nurses) in maternal and child health care institutions in this study were obtained from the 2017 China Health and Family Planning Statistical Yearbook and the 2018-2021 China Health and Health Statistical Yearbook. Regional per capita GDP and regional resident population data were from the 2017-2021 China Statistical Yearbook. The geographic area data was derived from the administrative division information of the Ministry of Civil Affairs of China. In particular, the data included in the study only included 31 provinces, municipalities, and autonomous regions in mainland China, excluding Hong Kong, Macau, and Taiwan.

### Setting

According to the level of regional economic development, mainland China is divided into four regions. Q4 regions refer to the regions with the highest per capita GDP, including Beijing, Shanghai, Jiangsu, Tianjin, Zhejiang, Fujian, Guangdong, and Shandong. Q3 regions refer to the regions with upper quartile per capita GDP, including Inner Mongolia, Chongqing, Hubei, Shaanxi, Liaoning, Hunan, Ningxia, and Jilin. Q2 regions refer to the regions with lower quartile per capita GDP, including Hainan, Anhui, Henan, Sichuan, Xinjiang, Jiangxi, and Qinghai. Q1 regions refer to the regions with the lowest per capita GDP, including Hebei, Tibet, Shanxi, Guangxi, Heilongjiang, Guizhou, Yunnan, and Gansu.

### Allocation level and fairness assessment

The health resource density index (HRDI) is used to measure the allocation level of health human resources in maternal and child health care institutions in different economic regions. The fairness of the allocation is evaluated according to the calculation results of the Gini coefficient (G) and Theil index (T). The research results reflect the development of China’s MCH human resources during the 13th Five-Year Plan period.

### Health resource density index

The HRDI is an indicator that comprehensively measures the level of health resource allocation by population and geographic area [25]. The calculation formula is:

$$HRDI=\frac{R_i}{\sqrt{A_i{P}_i}}$$

In the formula, *R*_*i*_ represents the MCH human resources owned by the *ith* region, *A*_*i*_ represents the geographic area of the *ith* region, and *P*_*i*_ represents the number of the resident population in the *ith* region. The larger the HRDI, the higher the allocation level of MCH human resources in the region.

### Gini coefficient

The Lorentz curve is often used in the medical and health field to explore the fairness of the allocation of health resources [24, 26]. However, the curve can only be displayed visually and can’t be quantified. Therefore, scholars introduce the G for quantitative evaluation. In essence, the G is the numerical embodiment of the Lorentz curve, with the same geometric meaning [27]. The calculation formula is:

$$G=1-\sum_{i=1}^{n-1}\left({\tau}_{i+1}-{\tau}_i\right)\left({\varphi}_{i+1}+{\varphi}_i\right)$$

In the formula, G is the Gini coefficient, *n* is the total number of regions, *τ*_*i*_ is the cumulative proportion of the population (geographical area) of the *ith* region in the country, and *φ*_*i*_ is the cumulative proportion of the *ith* region’s MCH human resources in the country. The G ranges from 0 to 1. The closer the G is to 0, the fairer the regional MCH human resources allocation is [28].

### Theil index

The T is derived from the concept of entropy in information theory and is used to measure the fairness of the allocation of health resources in a region [23]. At the same time, the T can be divided into the T between the groups (T_*inter*_) and the T within the group (T_*intra*_), which further reflects that the unfair allocation of regional resources is mainly caused by differences between groups or differences within groups [29]. Compared with the G, the T can examine the contribution of differences between groups and within groups to the total difference, making up for the limitation that it can only reflect the total difference [14]. The T calculation formula is:

$$T=\sum_{i=1}^n{\delta}_i\ln \left({\delta}_i/{\varepsilon}_i\right)$$

In the formula, *T* is the T, *n* is the total number of regions, *δ*_*i*_ is the proportion of the population (geographical area) of the *ith* region to the whole country, and *ε*_*i*_ is the proportion of human resources for MCH of the *ith* region to the whole country. The T ranges from 0 to 1. The smaller the value is, the fairer the regional MCH human resources allocation is [30].

The decomposition formula of the T is:

$$T={T}_{intra}+{T}_{inter}$$

$${T}_{intra}=\sum_{j=1}^k{\delta}_j{T}_j$$

$${T}_{inter}=\sum_{j=1}^k{\delta}_j\ln \left({\delta}_j/{\varepsilon}_j\right)$$

$${\omega}_{intra}={T}_{intra}/T$$

$${\omega}_{inter}={T}_{inter}/T$$

In the formula, *T*_*intra*_ is the T within the group, *T*_*inter*_ is the T between the groups, *k* is four regions with different economic levels, *δ*_*j*_ is the proportion of the population (geographical area) of the *jth* economic level region to the whole country, *T*_*j*_ is the T of the *jth* economic level region, *ε*_*j*_ is the proportion of MCH human resources in the *jth* economic level region to the whole country, *ω*_*intra*_ is the difference contribution rate within the group, and *ω*_*inter*_ is the difference contribution rate between the groups.

## Results

### Allocation level of health human resources in maternal and child health care institutions

**Fig. 1:**
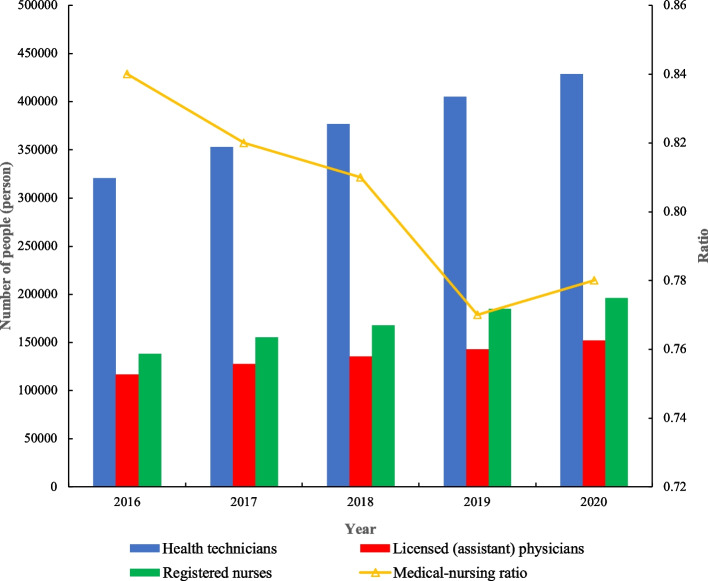
The allocation of health human resources in maternal and child health care institutions in mainland China from 2016 to 2020

Figure 1 showed the overall allocation of health human resources in maternal and child health care institutions in mainland China from 2016 to 2020. As of 2020, there were 428,809 health technicians in China’s maternal and child health care institutions, accounting for 83.31% of the total number of health staffs in that year. There were 152,076 licensed (assistant) physicians (accounting for 35.46% of health technicians) and 196,000 registered nurses (accounting for 45.71% of health technicians), with a medical-nursing ratio of 0.78. From 2016 to 2020, the number of health technicians, licensed (assistant) physicians, and registered nurses has been increasing year by year, with an average annual growth rate of 7.53, 6.88, and 9.12%, respectively. However, the medical-nursing ratio showed a fluctuating downward trend, from 0.84 in 2016 to 0.78 in 2020.

Table 1 showed the health resource density index of maternal and child health care institutions in mainland China in 2020. In general, the maximum HRDI of health technicians, licensed (assistant) physicians and registered nurses was in Guangdong, Beijing, and Guangdong, respectively, and the minimum was all in Tibet. Specifically, the distribution was different among different economic development level groups. In the Q4 regions, the maximum HRDI of the three types of MCH human resources was in Guangdong, Beijing, and Guangdong, and the minimum was all in Tianjin. Among the Q3 regions, the maximum HRDI of the three types of MCH human resources was all in Hunan, and the minimum was in Liaoning, Inner Mongolia, and Liaoning. Among the Q2 regions, the maximum HRDI of the three types of MCH human resources was all in Henan and the minimum was all in Qinghai. In the Q1 regions, Guangxi had the maximum HRDI of the three types of MCH human resources, and Tibet had the minimum.

**Table 1:** Health Resource Density Index of Maternal and Child Health Care Institutions in Mainland China in 2020

Group types	Health technicians				Licensed (assistant) physicians				Registered nurses			
	N/10^3^ population	N/10^3^ M^2^	HTDI	Rank	N/10^3^ population	N/10^3^ M^2^	LPDI	Rank	N/10^3^ population	N/10^3^ M^2^	RNDI	Rank
Q4 regions												
Guangdong	0.374	0.262	0.313	1	0.121	0.085	0.101	4	0.179	0.125	0.150	1
Beijing	0.268	0.357	0.309	2	0.113	0.150	0.130	1	0.112	0.150	0.130	3
Shandong	0.354	0.225	0.282	3	0.128	0.081	0.102	2	0.165	0.105	0.131	2
Zhejiang	0.327	0.211	0.263	4	0.126	0.081	0.101	3	0.146	0.094	0.117	6
Shanghai	0.102	0.401	0.202	10	0.039	0.153	0.077	7	0.047	0.186	0.094	10
Fujian	0.272	0.094	0.160	15	0.100	0.035	0.059	13	0.119	0.041	0.070	16
Jiangsu	0.159	0.123	0.140	17	0.067	0.052	0.059	14	0.066	0.051	0.058	19
Tianjin	0.070	0.081	0.075	24	0.039	0.046	0.042	21	0.014	0.016	0.015	27
Q3 regions												
Hunan	0.388	0.123	0.218	7	0.144	0.046	0.081	6	0.189	0.060	0.106	7
Hubei	0.378	0.114	0.208	9	0.129	0.039	0.071	11	0.189	0.057	0.104	8
Shaanxi	0.427	0.080	0.185	12	0.119	0.022	0.052	17	0.183	0.035	0.080	13
Chongqing	0.254	0.100	0.159	16	0.082	0.032	0.051	18	0.127	0.050	0.080	12
Ningxia	0.423	0.046	0.140	18	0.169	0.018	0.056	15	0.168	0.018	0.056	20
Jilin	0.201	0.025	0.071	25	0.091	0.011	0.032	24	0.076	0.010	0.027	24
Inner Mongolia	0.336	0.007	0.048	27	0.136	0.003	0.019	28	0.136	0.003	0.019	25
Liaoning	0.077	0.022	0.041	28	0.039	0.011	0.021	27	0.024	0.007	0.013	28
Q2 regions												
Henan	0.328	0.192	0.251	5	0.111	0.065	0.085	5	0.157	0.092	0.120	5
Jiangxi	0.409	0.109	0.211	8	0.139	0.037	0.072	10	0.195	0.052	0.101	9
Hainan	0.344	0.103	0.188	11	0.115	0.034	0.063	12	0.155	0.046	0.085	11
Sichuan	0.295	0.050	0.122	20	0.096	0.016	0.040	23	0.143	0.024	0.059	18
Anhui	0.164	0.072	0.108	22	0.066	0.029	0.044	20	0.068	0.030	0.045	22
Xinjiang	0.181	0.003	0.023	29	0.074	0.001	0.009	29	0.067	0.001	0.008	29
Qinghai	0.206	0.002	0.019	30	0.085	0.001	0.008	30	0.069	0.001	0.006	30
Q1 regions												
Guangxi	0.544	0.114	0.249	6	0.166	0.035	0.076	8	0.266	0.056	0.122	4
Hebei	0.289	0.114	0.181	13	0.120	0.047	0.075	9	0.114	0.045	0.072	15
Guizhou	0.346	0.074	0.160	14	0.114	0.024	0.053	16	0.163	0.035	0.075	14
Yunnan	0.379	0.046	0.132	19	0.117	0.014	0.041	22	0.178	0.022	0.062	17
Shanxi	0.239	0.052	0.112	21	0.096	0.021	0.045	19	0.098	0.021	0.046	21
Gansu	0.335	0.019	0.081	23	0.121	0.007	0.029	25	0.156	0.009	0.038	23
Heilongjiang	0.191	0.013	0.050	26	0.079	0.005	0.021	26	0.074	0.005	0.019	26
Tibet	0.105	< 0.001	0.006	31	0.036	< 0.001	0.002	31	0.034	< 0.001	0.002	31

*Note*: *HTDI* Health Technician Density Index, *LPDI* Licensed (Assistant) Physician Density Index, *RNDI* Registered Nursing Density Index

Table 2 showed the health resource density index of maternal and child health care institutions at different economic levels in mainland China from 2016 to 2020. In general, the HRDI of health technicians, licensed (assistant) physicians, and registered nurses all showed an upward trend year by year, with an average annual growth rate of 7.25, 6.60, and 8.83%, respectively. In terms of intra-group comparison, the HRDI of the three types of MCH human resources allocation showed a ladder distribution. It showed the Q4 regions hit the highest HRDI, while the Q2 regions and the Q1 regions obtained the lowest HRDI. The HRDI of the Q3 regions was between the highest and the lowest.

**Table 2:** Health resource density index of maternal and child health care institutions in different economic levels in mainland China from 2016 to 2020

Personnel category	2016	2017	2018	2019	2020
Health technicians					
entire country	0.088	0.096	0.103	0.110	0.116
Q4 regions	0.190	0.206	0.218	0.235	0.238
Q3 regions	0.088	0.096	0.099	0.106	0.112
Q2 regions	0.066	0.072	0.077	0.083	0.090
Q1 regions	0.071	0.081	0.089	0.095	0.103
Licensed (assistant) physicians					
entire country	0.032	0.035	0.037	0.039	0.041
Q4 regions	0.066	0.072	0.077	0.084	0.086
Q3 regions	0.033	0.036	0.037	0.038	0.040
Q2 regions	0.023	0.025	0.026	0.028	0.031
Q1 regions	0.028	0.031	0.033	0.034	0.036
Registered nurses					
entire country	0.038	0.042	0.046	0.050	0.053
Q4 regions	0.084	0.092	0.098	0.107	0.109
Q3 regions	0.038	0.041	0.043	0.049	0.052
Q2 regions	0.030	0.033	0.036	0.039	0.042
Q1 regions	0.028	0.034	0.039	0.042	0.046

### Quality structure of health human resources allocation in maternal and child health care institutions

Table 3 showed the quality structure of health human resources in maternal and child health care institutions in mainland China from 2016 to 2020. In terms of age, the majority of health technicians and registered nurses were 25-34 years old, and the proportion was increasing, while the proportion of licensed (assistant) physicians was mainly concentrated in 35-44 years old, and the proportion was decreasing. In terms of educational background, health technicians and registered nurses were mainly junior college, while licensed (assistant) physicians were mostly undergraduate. Overall, the proportion of the three types of MCH human resources with undergraduate or above showed an increasing trend. In terms of job titles, the majority of health technicians and licensed (assistant) physicians were at the level of doctor, while registered nurses were concentrated at the level of assistant nurse.

**Table 3:** Quality structure of health human resources in maternal and child health care institutions in mainland China from 2016 to 2020

Category	Health technicians /%		Licensed (assistant) physicians /%		Registered nurses /%	
	2016	2020	2016	2020	2016	2020
Age						
<25	8.6	6.0	0.1	0.3	13.8	9.2
25~	38.6	39.7	21.9	23.3	46.5	50.2
35~	29.1	28.4	38.9	34.2	24.2	25.2
45~	19.8	19.2	31.2	29.7	14.0	12.5
55~	2.3	4.8	4.0	8.5	1.0	2.4
≥ 60	1.7	1.9	3.9	4.0	0.3	0.5
Education						
Postgraduate	2.5	3.8	5.0	8.7	0.1	0.1
Undergraduate	29.4	39.7	44.9	54.1	15.1	28.3
Junior college	43.2	40.9	35.2	28.2	51.2	51.1
Technical secondary school	24.1	15.1	14.3	8.6	33.1	20.2
High school and below	0.9	0.4	0.5	0.2	0.6	0.2
Job title						
Professor of treatment/nursing	1.2	1.8	3.1	4.3	0.2	0.3
Associate professor of treatment/nursing	6.3	7.8	14.1	15.9	2.4	3.5
Doctor/nurse-in-charge	23.1	22.7	36.8	32.7	18.0	18.5
Doctor/nurse practitioner	29.8	30.9	36.1	34.9	26.4	28.6
Assistant doctor/nurse	29.8	29.4	6.0	7.6	45.5	43.3
Unknown	9.9	7.4	3.9	4.6	7.5	5.7

### Fairness of health human resources allocation in maternal and child health care institutions

#### Gini coefficient measurement results

**Fig. 2:**
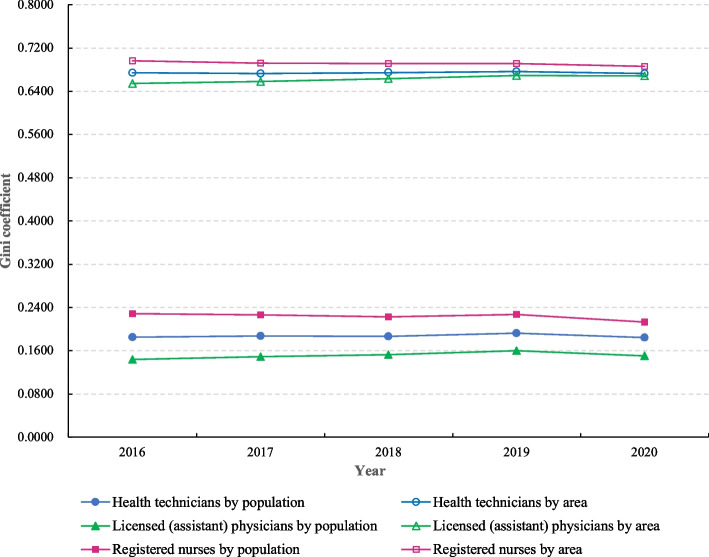
The Gini coefficient of the allocation of health human resources in maternal and child health care institutions in mainland China from 2016 to 2020

Figure 2 showed the G of the allocation of health human resources in maternal and child health care institutions in mainland China from 2016 to 2020. The G of health technicians, licensed (assistant) physicians and registered nurses by population were 0.1844-0.1924, 0.1435-0.1599, and 0.2131-0.2285, respectively. The allocation of health technicians and licensed (assistant) physicians was fair, while the allocation of registered nurses was relatively fair. If allocated according to the geographical area, the G of health technicians, licensed (assistant) physicians and registered nurses were 0.6730-0.6765, 0.6547-0.6689, and 0.6858-0.6965, respectively. All three types of resources were in a very unfair state. In addition, it could also be found that the G of the three types of MCH human resources allocated by population were all smaller than those allocated by geographical area. Notably, the G by population and by geographic area for health technicians and registered nurses showed a slight downward trend over the 5 years, while the opposite was true for licensed (assistant) physicians.

#### Theil index calculation results

Table 4 showed the T of the allocation of health human resources in maternal and child health care institutions in mainland China from 2016 to 2020. The total T of health technicians, licensed (assistant) physicians, and registered nurses by population were 0.027-0.035, 0.016-0.022, 0.044-0.052, respectively, and the total T indices by geographical area were 0.586-0.617, 0.538 ~ 0.593, 0.654 ~ 0.675, respectively. Overall, the total T of the three types of MCH human resources allocated by geographic area was significantly greater than that allocated by population. In addition, the total T of the three types of MCH human resources allocated by population and geographical area showed an upward trend.

**Table 4:** The T of the allocation of health human resources in maternal and child health care institutions in mainland China from 2016 to 2020

Personnel category	Configured by population					Configured by geographic area				
	2016	2017	2018	2019	2020	2016	2017	2018	2019	2020
Health technicians										
T	0.027	0.029	0.032	0.035	0.033	0.586	0.586	0.609	0.613	0.617
T_*inter*_	0.001	0.001	0.001	0.000	0.001	0.091	0.088	0.086	0.087	0.083
T_*intra*_	0.027	0.029	0.031	0.034	0.032	0.496	0.498	0.523	0.526	0.533
Licensed (assistant) physicians										
T	0.016	0.018	0.020	0.022	0.021	0.538	0.546	0.576	0.586	0.593
T_*inter*_	0.002	0.002	0.001	0.001	0.001	0.085	0.086	0.086	0.090	0.088
T_*intra*_	0.015	0.016	0.019	0.021	0.020	0.453	0.460	0.489	0.495	0.505
Registered nurses										
T	0.044	0.046	0.048	0.052	0.048	0.657	0.654	0.675	0.669	0.674
T_*inter*_	0.001	0.000	0.000	0.000	0.001	0.095	0.091	0.086	0.087	0.083
T_*intra*_	0.043	0.045	0.048	0.052	0.047	0.562	0.563	0.589	0.583	0.591

By decomposing the total T, it could be found that the T_*intra*_ of health technicians, licensed (assistant) physicians, and registered nurses by population and by geographic area was greater than T_*inter*_. The contribution rates of the T_*intra*_ of the three types of MCH human resources allocated by population to the total T were 97.22 to 98.58%, 90.13 to 95.95%, and 98.55 to 99.53%, respectively. If the three types of MCH human resources were allocated according to the geographical area, the contribution rates of the T_*intra*_ to the total T were 84.55-86.47%, 84.15-85.22% and 85.56-87.69%, respectively.

Table 5 showed the contribution rate of the T to the total T of the allocation of health human resources in maternal and child health care institutions at different economic levels in mainland China from 2016 to 2020. The contribution rates of the T of health technicians and registered nurses to the national total T were all in the Q2 regions < Q1 regions < Q3 regions < Q4 regions, while the licensed (assistant) physicians were in the Q1 regions < Q2 regions < Q3 regions < Q4 regions. If allocated according to the geographical area, the contribution rates of the T of the three types of MCH human resources to the national total T were all in the Q4 regions < Q3 regions < Q2 regions < Q1 regions.

**Table 5:** The contribution rate of the T of the health human resources allocation of maternal and child health care institutions in different economic levels in mainland China to the total T from 2016 to 2020

Personnel category	Contribution rate by population allocation /%					Contribution rate by geographic area /%				
	2016	2017	2018	2019	2020	2016	2017	2018	2019	2020
Health technicians										
Q4 regions	35.99	36.54	37.33	36.01	35.89	0.42	0.43	0.43	0.45	0.41
Q3 regions	27.49	28.36	28.16	31.28	32.84	11.98	12.06	11.67	11.75	11.76
Q2 regions	16.46	16.07	16.96	14.64	13.88	36.08	35.17	34.33	34.62	35.22
Q1 regions	18.15	17.16	15.71	16.65	14.62	36.07	37.25	39.46	38.98	39.08
Licensed (assistant) physicians										
Q4 regions	35.43	36.45	37.54	36.79	37.23	0.38	0.36	0.36	0.36	0.34
Q3 regions	30.98	31.22	29.31	31.87	33.78	11.18	11.11	10.63	10.51	10.72
Q2 regions	15.10	13.20	15.03	13.84	13.33	35.47	34.43	33.10	33.11	33.60
Q1 regions	8.62	10.63	11.20	13.20	11.61	37.12	38.35	40.95	40.59	40.56
Registered nurses										
Q4 regions	28.77	32.01	33.63	33.41	34.94	0.43	0.48	0.49	0.53	0.48
Q3 regions	31.42	31.44	30.41	32.86	32.85	12.76	12.50	11.98	12.33	12.26
Q2 regions	16.06	16.94	18.17	15.34	14.50	36.22	36.35	35.60	36.05	36.27
Q1 regions	22.33	19.05	17.25	17.92	16.25	36.15	36.78	39.14	38.15	38.68

## Discussion

This study uses longitudinal data from 2016 to 2020, divides regions according to regional economic levels, and explores the allocation and fairness of health human resources in maternal and child health care institutions in mainland China. The results of the analysis can reveal the remaining problems in the current development, help rationally allocate the human resources for MCH, provide more equitable and accessible MCH services, and promote the high-quality development of the cause of MCH. To the best of our knowledge, this is the first study on the allocation of MCH human resources using nationwide longitudinal data after the adjustment of the new fertility policy.

In terms of allocation level, the allocation level of MCH human resources in mainland China has been continuously improved. From 2016 to 2020, the number of health technicians, licensed (assistant) physicians, and registered nurses in maternal and child health care institutions showed an increasing trend year by year. By the end of 2020, health technicians have accounted for 83.31% of the total number of health staffs, and the ratio of medical care to nurses has reached 1:1.28, which has completed the “China Medical and Health Service System Planning Outline (2015-2020).” The staff ratio is not less than 80% of the total number and the mission target of a medical-to-care ratio of 1:1.25 [31]. This shows that the Chinese government’s efforts to improve the MCH service system are very effective.

However, there are differences in the level of MCH staffing in regions with different economic development levels. In 2020, the HRDI of health technicians and registered nurses in Guangdong would be about 52 times and 75 times that of Tibet, respectively, while the HRDI of licensed (assistant) physicians in Beijing would be about 65 times that of Tibet. In the five-year comparison, the HRDI in the Q4 regions were the highest, followed by the Q3 regions, and the lowest in the Q2 regions and the Q1 regions. Analyzing the reasons, on the one hand, may be related to the level of economic development and population density between regions. The more developed the regional economy and the higher the population density, the greater the investment in MCH resources. On the other hand, regions with better economic development are more attractive to health professionals and have more development opportunities [32]. Therefore, in the future, government health investment will be supposed to focus on balancing regional differences, and appropriately tilt towards regions with lower levels of economic development. At the same time, a talent incentive policy will be formulated to encourage health staffs to go to poor areas with relatively scarce health resources [33].

We find that when analyzing the allocation of health human resources, few studies have explored the quality structure of allocation. This study adds to this section. The results showed that health technicians and registered nurses in maternal and child health care institutions in mainland China were mainly junior college while licensed (assistant) physicians were mainly undergraduate, which was similar to the findings of Ren Z et al. [34]. A previous study found that in 2005, 67.2% of licensed (assistant) physicians and 97.5% of registered nurses in China had only junior college or technical secondary school [35]. Compared with the national average, the education level of MCH staffs has been greatly improved, and they can provide higher quality MCH services. In addition, the job titles of health technicians and licensed (assistant) physicians were mainly at the level of doctor, and registered nurses were concentrated at the level of assistant nurse. The WHO has recommended that the ratio of job titles for health staffs should be 1:3:1 in the ratio of senior, intermediate, and junior [36]. At present, there is still a gap in this standard. It is suggested to improve the continuing education mechanism of MCH staffs, reasonably standardize the scale and structure of staffs training, and continuously improve their knowledge.

In terms of allocation fairness, this study showed that the allocation of MCH technicians, licensed (assistant) physicians, and registered nurses in mainland China was fair by population, but not by geographical area. This conclusion is consistent with previous research [27, 37, 38]. The reason may be that government health departments usually use the number of health resources per 1000 population as the standard for regional planning and allocation, and pay less attention to the geographic availability of health resources [39]. It should be noted that the results were still meaningful in an international context. Similar situations also existed in other countries, such as Kenya, Mexico, Vietnam, Japan, Mongolia, etc. [37, 40, 41]. The unfair geographical allocation of health resources would restrict the rational allocation and management of health resources, and ultimately affect the fairness and accessibility of health services. Of course, the relationship between population size and geographical area also needs to be carefully considered. If fewer people live in remote rural areas, this may be a more efficient way to allocate resources. Therefore, it is suggested that when formulating MCH care planning in the new period, the two factors of service population and geographical area should be considered comprehensively, and regional differences should be balanced, so as to continuously meet the needs of residents for MCH care services. On the other hand, other countries can learn from the Chinese model such as setting the total target of human resource development for MCH and conducting regular assessment.

The T is also decomposed in this study. The results showed that the unfairness in the allocation of MCH human resources was mainly caused by differences within regions, which was consistent with the results of previous studies [14, 42, 43]. The contribution rate of different economic regions to the national total T was further analyzed. The study found that if allocated by population, the main reasons for the unfairness in the allocation of MCH human resources were the Q2 regions and the Q1 regions. It was worth noting that if the allocation was based on geographical area, the Q4 regions were the main reason for the unfair allocation. It is not difficult to understand that Tibet, Xinjiang, and other places are sparsely populated and have a large service radius, while Beijing, Shanghai, and other places are on the contrary, and the economic development levels of the two are also far from each other. At this time, the allocation of MCH resources according to the population is obviously in favor of the latter, geographically in favor of the former. Therefore, it is suggested that policymakers need to fully understand the impact of intra-regional differences on the allocation of MCH human resources, increase financial support for regions with medium and low GDP per capita, introduce corresponding employment guidance policies, and attract more outstanding health professionals.

This study has some limitations. First, the difference between urban and rural areas is an important factor contributing to health unfairness in China. However, the research data are from the China Health Statistical Yearbook, and there is a lack of urban-rural disaggregated data at the level of maternal and child health institutions. Second, the research subjects are only included in the 31 provinces, municipalities, and autonomous regions in mainland China, excluding Hong Kong, Macau, and Taiwan. The medical and health systems in these regions are somewhat different from those in mainland China. In the future, we can further explore their impact on the fairness of China’s overall MCH human resources allocation. Third, the research objects are divided into regions based on the level of economic development, and the contribution rates of differences between groups and within groups to the total T are analyzed to reflect the overall fair impact of economic and non-economic factors. Nevertheless, the specific impact of economic and non-economic factors on the equity of MCH staffing has not been studied. Finally, this study only discusses the fairness of allocation and ignores the efficiency of allocation. In the future, evaluation can be made on this basis, so that the allocation of MCH human resources can take into account both fairness and efficiency.

## Conclusions

This study focuses on the distribution of health human resources in maternal and child health care institutions in mainland China and the fairness of their distribution. Although the Chinese government has made great efforts in MCH work in the past, our research shows that there are still differences in the distribution of MCH staffs in different economic development regions in China, and the fairness of allocation by population is better than allocation by geographical area. In addition, the quality structure of MCH staffs is still far from the standard recommended by the WHO. In the future, policymakers should especially focus on considering the impact of intra-regional differences on the allocation of MCH human resources, and continuously improve the geographic accessibility of MCH services.

## Data Availability

The data for this study came from: a). China National Health Commission official website (http://www.nhc.gov.cn/wjw/index.shtml), b). China National Bureau of Statistics official website (http://www.stats.gov.cn/) and c). China Ministry of Civil Affairs official website (http://www.mca.gov.cn/).

## Abbreviations

GDP: Gross Domestic Product
HRDI: Health resource density index
G: Gini coefficient
FA: Fairness assessment
T: Theil index
>T_*intra*_: Theil index within the group
T_*inter*_: Theil index between the groups
MCH: Maternal and child health
WHO: World Health Organization
HTDI: Health Technician Density Index
LPDI: Licensed (Assistant) Physician Density Index
RNDI: Registered Nursing Density Index
